# Editorial: Women and substance use: Specific needs and experiences of use, others' use and transitions towards recovery

**DOI:** 10.3389/fpsyt.2022.1078605

**Published:** 2022-12-13

**Authors:** Lucy Webb, Sarah Fox, Anette Skårner, Guilherme Messas

**Affiliations:** ^1^Faculty of Health, Psychology and Social Care, Manchester Metropolitan University, Manchester, United Kingdom; ^2^Faculty of Arts and Humanities, Manchester Metropolitan University, Manchester, United Kingdom; ^3^Department of Social Work, Faculty of Social Sciences, University of Gothenburg, Gothenburg, Sweden; ^4^Faculty of Medical Sciences, Santa Casa of São Paulo, São Paulo, Brazil

**Keywords:** women, female substance use, women's needs, women's experience, substance use, women and girls' substance use services

Problematic substance use among women is increasing, with global prevalence data indicating that 46 million women have an alcohol use disorder (1), the highest prevalence of which is located in the European region (2). Reports from the European Monitoring Centre for Drugs and Drug Addiction (EMCDDA) also show that women make up a quarter of people with illicit problematic drug use in Europe (3). Globally, approximately a third of all drug users are women, and a fifth of injecting drug users are women (4).

Historically, societal and medical responses to substance use issues were shaped based on men as the major protagonists, with women's use only acknowledged when it impacted their care-giving role (5, 6). Feminist perspectives have more recently identified how women experience drug and alcohol use, the type of substances they use, the spaces they consume substances, and their treatment and support needs (7). However, researchers and practitioners need to continue the dialogue on women's substance use in order to expand knowledge, challenge prejudices, and learn to support women in a way that is specific to their needs.

Indeed, women are often more affected by substance use than men, and more affected negatively by others' use (4). They commonly experience multiple types of disadvantage and trauma, including violence and abuse, sex-work, poverty, and mental ill-health (8). There are also unique physical health risks including breast cancer, ovulation and menstrual difficulties, early menopause, and fertility issues, as well as risks to the child in pregnancy (9, 10). Women from minority ethnic groups face additional challenges and barriers (11).

Trauma is frequently a factor in substance use among both men and women, typically used to manage the experiences of trauma (12). However, as this collection of studies demonstrates, women face a unique set of additional gender-based traumas that can further influence their use of drugs or alcohol, such as domestic abuse, coercive and controlling behavior and being forced to exchange sex for substances, often for their male perpetrators (13, 14).

Women also face unique barriers to support, and their needs may be unmet by existing services (15) because treatment and support are delivered in ways that do not meet the lived reality of their lives (6, 16, 17). Recently, advocates for substance-using women have called for a more gender-sensitive response to drug policy and support that addresses “the harms directly related to drugs and drug use but also the social and cultural determinants of drug use and health and law enforcement policies” [(5), p. 17]. This is reflected in the papers presented in this Research Topic on women and substance use.

This collection of international studies highlights the unique challenges experienced by women who use substances, and demonstrates the gender-specific contexts of their substance use including the specific risks they face, and the social, health and policy factors that impact their use, and access to treatment and support.

The evidence reported by Guy et al. illustrates the particular vulnerability of women to HIV, where women face barriers to pre-exposure prophylaxis. These barriers are linked to homelessness, sexual violence, being in a drug-using relationship, and having a child-rearing role which may contribute to reluctance to engage with services. Pedersen et al. highlight an additional risk to HIV among sex-workers using amphetamines, presenting a further harm reduction risk because of the disinhibiting effect and increased risk-taking. Both these studies also highlight problems of the lower status of women to men, especially for sex workers, and, for Pedersen et al., particularly in countries with strong cultural gender morals and criminalization of sex work.

Vulnerability to gender-based violence (GBV) is underlined in several papers. For Pedersen et al., in comparison to men, women are more likely to have an intimate partner who uses substances, be dependent on their partner for supply, and experience more physical and psychological violence within such relationships. Moir et al. report that intimate partner violence is often hidden among women affected by substance use, and may only be detected during tertiary-level risk assessment. Morton et al.'s study highlights the degree of parental substance use and adverse childhood experiences among substance-using women experiencing domestic violence. These studies suggest an iceberg of hidden GBV cases among women substance users who need proactive screening to ensure the right support is offered.

In comparison to men, Webb et al.'s study of drug-related deaths also shows women are disproportionately at risk from polydrug use and more likely than men to increase their risk by using prescribed methadone and benzodiazepines alongside illicit substances. There may also be trends in age differences for women, with smaller gender differences in risky use among younger, recreational drug use fatalities, and greater risky polydrug use among older female fatalities. Addressing aging needs among women in recovery is also highlighted by Shaw et al., who found that the emergence of physical and mental symptoms for middle-aged women in recovery requires more attention from services.

Several studies underline gender inequality for women affected by substance misuse. Russell et al. report substance-using mothers six times more likely to have children removed than substance-using fathers, and more likely to attempt suicide that women substance users without children. Richert takes a different perspective of gender difference by examining how women navigate a role in the drug economy in which men have control over income and drugs supply. His study shows women using their sexuality or adopting a professional or more masculine persona to reduce risk in securing income and drugs. Bäcklin also identifies an existing “macho culture” in peer support provision that presents unequal support for women, but also illustrates how proactive efforts to represent and empower women's voices can be effective.

Some of these studies also suggest a self-medication role for women's substance use. The study by Pedersen et al., suggests use of amphetamines in sex work to improve performance, but also to dissociate from the work, and this is also suggested by Tractenberg et al.'s study showing crack cocaine use among Brazilian women was a dissociative coping strategy for negative life experiences. Webb et al. highlight women's disproportionate high risk polydrug use that includes prescribed anti-anxiety and sedative medications, and Dahlberg et al. report young substance-using women and girls more likely to have co-occurring psychiatric problems and experiences of trauma than males on entering treatment.

Several studies in this issue investigate more gender-sensitive interventions to better address women's specific needs. In Morton et al.'s study, women using a domestic violence and substance use service had high rates of existing adverse childhood experiences (ACEs). They suggest that routine enquiry about ACEs for this population starts a process of trauma-informed service responses. Moir et al. go further to recommend that more integrated service working for women substance users experiencing GBV would ensure access to specialist support. Similarly, integrated service approaches are recommended by Petzold et al. to include access to stable housing to increase engagement with services. Harwin et al. argue that the introduction of family drug and alcohol courts may avoid current organizational silo practices through offering more holistic interventions to better support women and children through the complexities of DV, substance use and child maltreatment. Dahlberg et al. also indicate that specific multi-dimensional and longer intervention times for young women and girls would be a more responsive approach to the greater prevalence of mental ill health and trauma among young women and girls.

Overall, gender-responsive service approaches for women are indicated by these studies, and Schamp et al.'s recommendations go further, suggesting a transformative approach that goes beyond the more reactive gender responsiveness. They argue that this could activate policy makers toward holistic strategies to address the contextual factors associated with women's substance use.

This collection underlines and adds to the evidence of the specific issues facing women substance users. Studies here show that, beyond substance-related stigma, women around the world face different and multiple pressures to use substances, and encounter structural barriers to reducing harm and accessing appropriate treatment. Societal norms of femininity and motherhood place women in a vulnerable position within their societies, exposing them to GBV, judgement and disempowerment. Many studies illustrate the whole-system mechanisms that maintain this vulnerability, and highlight the need for gender-sensitive approaches in policies and practice. Indeed, while services may adopt specific provision for women's needs, this may be seen as an add-on to male-oriented services that does not address the wider context. As suggested in Schamp et al.'s article, policymakers may need to embrace a more gender-transformative approach across services and, arguably, focus on gender justice as much as gender equity.

## Author contributions

SF and LW contributed to the drafting. AS and GM contributed to the review process and finalization of the manuscript. All authors contributed to the article and approved the submitted version.
